# Sub-Decadal Resolution in Sediments of Late Miocene Lake Pannon Reveals Speciation of *Cyprideis* (Crustacea, Ostracoda)

**DOI:** 10.1371/journal.pone.0109360

**Published:** 2015-04-22

**Authors:** Frank Gitter, Martin Gross, Werner E. Piller

**Affiliations:** 1 Department for Geology & Palaeontology, Universalmuseum Joanneum, Weinzöttlstrasse 16, 8045, Graz, Austria; 2 Institute of Earth Sciences, University of Graz, NAWI Graz, Heinrichstrasse 26, 8010, Graz, Austria; Universität Göttingen, GERMANY

## Abstract

Late Miocene "Lake Pannon" (~11.3 Ma) was a remnant of the Central Paratethyan Sea. Successive freshening and constantly changing environmental conditions, like oxygenation, nutrition and substrate led to a well-documented radiation in molluscs and ostracods. Among ostracods (small crustaceans), *Cyprideis* is one of the most common genera in "Lake Pannon", as well as in several other ancient lakes, showing numerous adaptations and speciations. Here, we present high-resolution data from an early transgression of "Lake Pannon" in the Eastern Styrian Basin (SE Austria). Mataschen clay pit is in the focus of geologic and paleontologic research since 20 years and its geologic and paleoecologic evolution is well-documented. We drilled five cores covering a ~2.3 m long section and completely sampled it in 5-mm thick intervals to reconstruct minute changes in the ostracod fauna over a transgression of a brackish water body. The dominant genus, *Cyprideis*, is represented by three species *C*. *mataschensis*, *C*. *kapfensteinensis* and *C*. ex gr. *pannonica*. Through morphometric analyses we highlight the variance of each taxon and suggest that there is no direct ecologic control on size or shape. Furthermore, we found a second, co-occurring morphotype of *C*. *kapfensteinensis* which is directly related to an elevation of salinities above 13 psu. The presence of two intermediate specimens between the two morphotypes in the sample directly below the first appearance of *C*. *kapfensteinensis* B leads us to the conclusion that we are facing a speciation event leading to four sympatric species of *Cyprideis*.

## Introduction

Ostracods of the genus *Cyprideis* Jones, 1857 [1] are euryhaline, benthic crustaceans typically occurring in coastal brackish waters. Their first appearance in the fossil record possibly dates back to the Upper Oligocene [2–4]. Recent *Cyprideis torosa* (Jones, 1850) [5] is known to cope with a wide range of salinities, occurring from slightly brackish to hypersaline environments (~2–120 psu; [6, 7]. Populations reach densities of up to 330,000 ind./m^2^ [7, 8] and are also able to withstand short periods of hypoxia [9, 10]. *Cyprideis torosa* has been the focus of many ecologic studies over the years. One of the main topics in research concerning *C*. *torosa* was the occurrence of nodes on the outside of the valves which is related to salinity and Ca^2+^ availability of the host water [3, 11–14]. Frenzel et al. [15] performed microcosm experiments and related noding to salinities <14 psu and <7 psu under Ca^2+^ deficiency. Analyses of the shape of sieve pores are another indicator for paleosalinities as the number of round sieve-pores increases with decreasing salinity [16, 17]. The size of the carapace seems to be dependant of the salinity [18] or nutrient supply [11]. Boomer and Frenzel [19] reported a decrease in size above salinities of 8–9 psu. This coincides with a switch of the osmoregulation in *C*. *torosa* from hyper- to iso-osmoregulation at around 8–9 psu [20, 21].

Apart from this wide range of adaptabilities, *Cyprideis* is also known for its diverse speciation in ancient lake systems. In African long-lived Lake Tanganyika, *Cyprideis* evolved into at least 23 endemic species and split in 6 genera over the last 9–12 million years [22–24]. Similar speciation events are also seen in the Miocene of the Western Amazon Basin [25–27], in the Late Miocene of the Pannonian Basin [2, 28, 29], Turkey [30] and the Paleomediterranean [31, 32].

**Fig 1:**
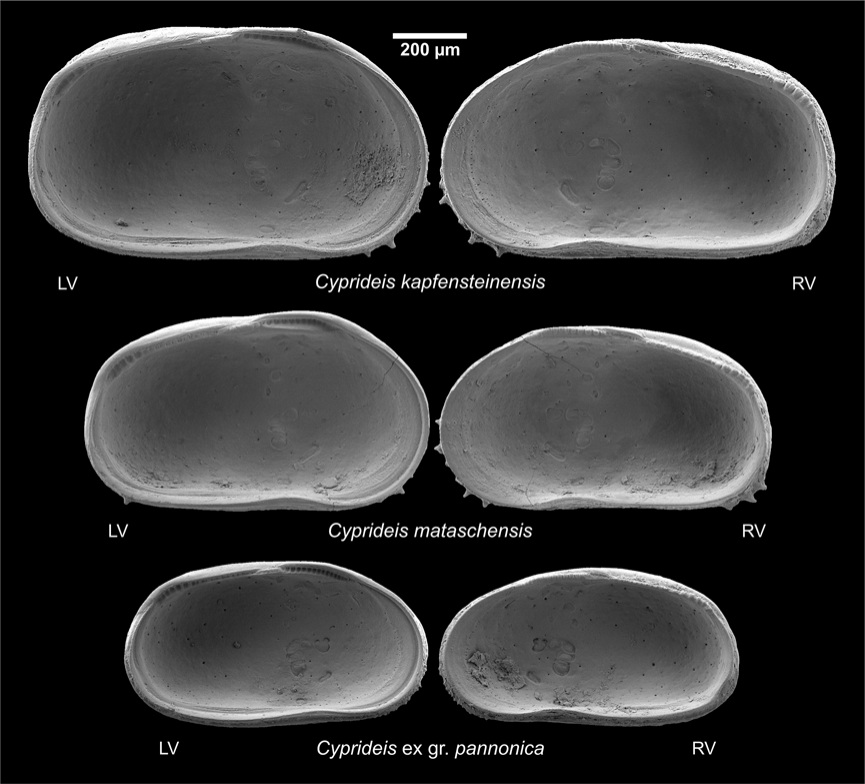
*Cyprideis* species from Mataschen. Differences in the hinge structure, outline and size between female *C*. *kapfensteinensis*, *C*. *mataschensis* and *C*. ex gr. *pannonica*. LV indicates left valves, RV indicates right valves.

The work presented here highlights the variability of two closely related *Cyprideis* species (*C*. *kapfensteinensis* Gross, 2008 [33] and *C*. *mataschensis* Gross, 2008 [33]) from the early phase of "Lake Pannon" (~11.3 Ma). While very similar in shape and ornamentation they can be clearly distinguished by their hinge (Fig 1), the number of posteroventral spines in the left valve and their size. A third co-occurring species, *C*. ex gr. *pannonica* (Méhes, 1908) [34], is well separated by its smooth ornamentation, more elongated shape with the maximum height further posterior, the lack of posteroventral spines and their smaller size [33]. Here, we subdivide *C*. *kapfensteinensis* into two morphotypes A and B. Whereas *C*. *kapfensteinensis* A represents *C*. *kapfensteinensis sensu* [33], *C*. *kapfensteinensis* B shows a well-developed sulcus, a somewhat coarser ornamentation and a slightly different hinge structure. The anterior hinge element in *C*. *kapfensteinensis* B is slightly broader compared to *C*. *kapfensteinensis* A.

While *Cyprideis* is known for its high intraspecific variability [3], each taxon varies only within a certain range of its general habitus [2]. Therefore, a taxonomic distinction based on its shape is possible. While the human eye is very well able to differentiate between shapes, a quantitative way to distinguish those shapes can help a lot to visualize taxonomic disparities [35]. Analyses of the outlines (*i*.*e*. shapes) offer the possibility to objectively dissect the differences between ostracod species [32, 36, 37]. Geometric morphometrics are widely used among ostracod taxonomists [38–41]. Most common are landmark analyses [38, 42, 43] or Fourier analyses of the outline [35, 44, 45], but during the last few years B-spline analyses of the outline became more popular among ostracodologists [32, 33, 46, 47]. Here we show the intra- and inter-specific variability of two closely related species (*C*. *mataschensis* and *C*. *kapfensteinensis*) and two morphotypes of *C*. *kapfensteinensis* (*C*. *kapfensteinensis* A and *C*. *kapfensteinensis* B) during an early transgressive phase of Lake Pannon and the relation of their morphologic variability in response to ecologic changes.

### Geologic Setting

**Fig 2:**
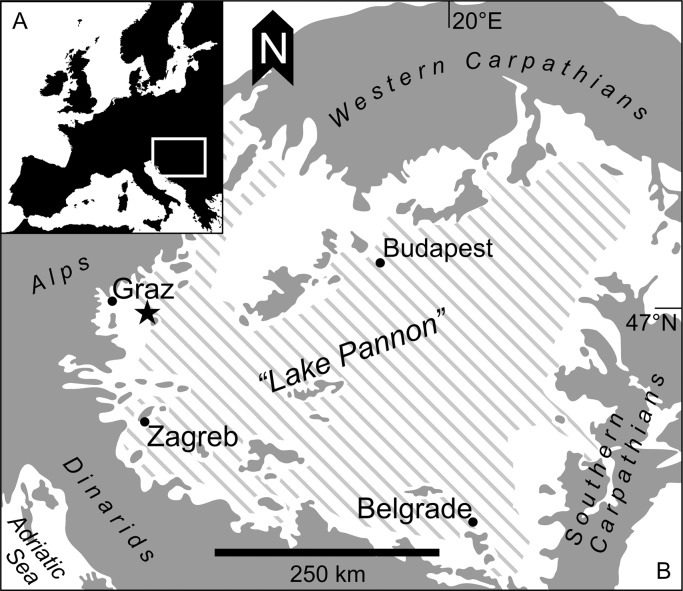
Geographic overview of the Pannonian Basin. a) Geographic location of the Pannonian Basin b) Extension of Lake Pannon in the Pannonian Basin in the early Late Pannonian and location of the Mataschen clay pit.

**Fig 3:**
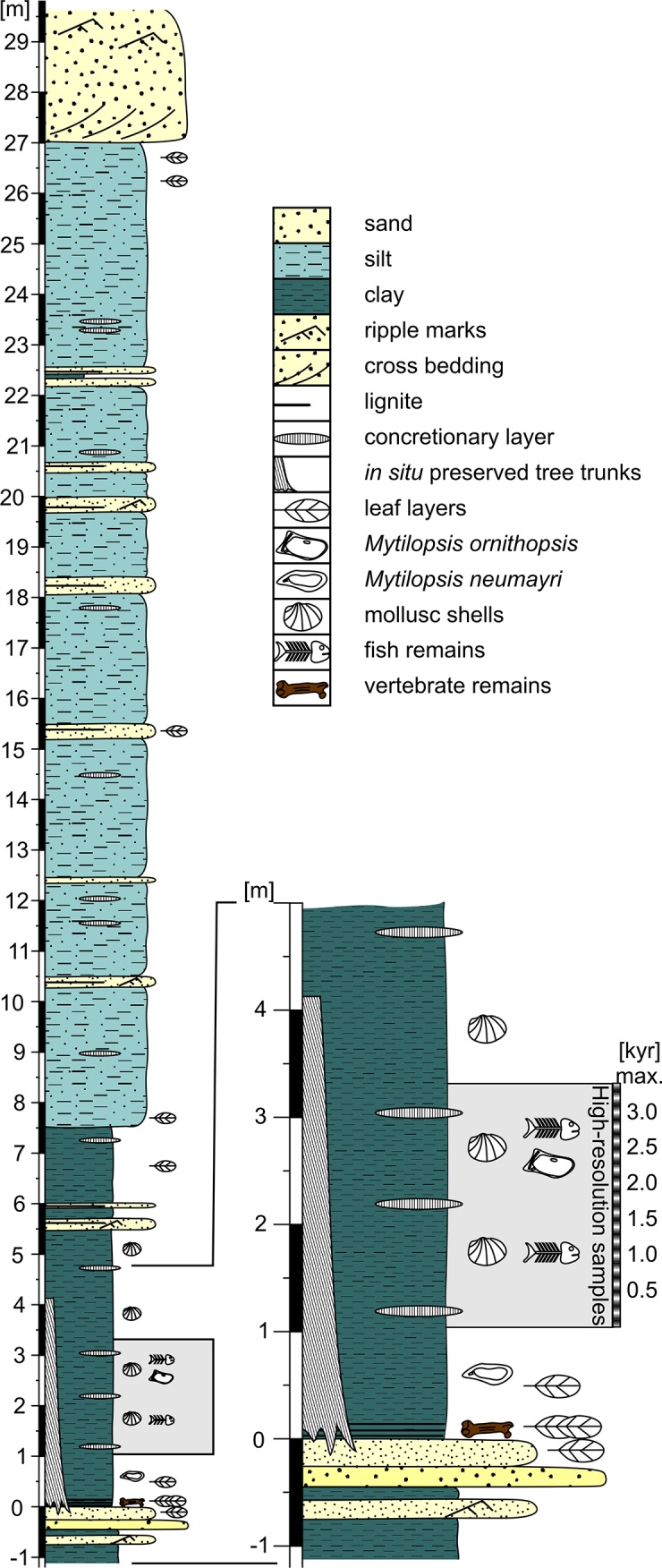
Section of the Mataschen clay pit. Lithological column of the Mataschen clay pit and cut-out of the section comprising the high-resolution samples discussed herein (after Gross et al. 2011).

The Mataschen clay pit is situated approx. 40 km SE of Graz (SE, Austria, 15°57′16″E/46°54′15″N; Fig 2). The clay pit exposes a *c*. 30 m thick succession of clay and silt with intercalated sands (Feldbach Formation, [48]). On top, unconformably overlying cross-bedded sands of the Paldau Formation are exposed (Fig 3). Based on paleontologic and paleomagnetic data the section could be dated as 11.308–11.263 Ma equating to the early Late Miocene [49]. The timeframe of less than 45k years for the accumulation of the whole succession means an estimated sedimentation rate between 0.7 and 1.4 mm/yr [49]. Thus, the 5-mm thick samples from the high-resolution interval, lead to a very fine temporal resolution of less than one decade per sample.

At the base of the Mataschen clay pit, in-situ preserved *Glyptostrobus* tree trunks indicate a swampy environment. A rapidly rising water level led to the drowning of the swamp and the establishment of a nearshore brackish lake fauna including fishes, bivalves and ostracods (see [50]).

## Material and Methods

Five sediment cores (each ~0.5 m long, 100 mm in diameter) were drilled near the base of the *c*. 30 m thick succession of the Mataschen clay pit, SE Austria. The cores were cut in half and split into 447 samples each 5-mm thick. Samples were treated with diluted hydrogen peroxide (H_2_O: H_2_O_2_ = 5: 1), washed and sieved through standard sieves (500/250/125/63 μm) and dried at 40°C for at least 24 hours. Fractions >250 μm were picked completely. Smaller fractions were only briefly scanned; they contained mostly broken valves and early instars (A-3 and younger) of ostracods. The samples are stored in micropaleontologic slides at the Universalmuseum Joanneum (Dept. Geology & Palaeontology), Graz.

No permits were required for the described study, which complied with all relevant regulations.

**Fig 4:**
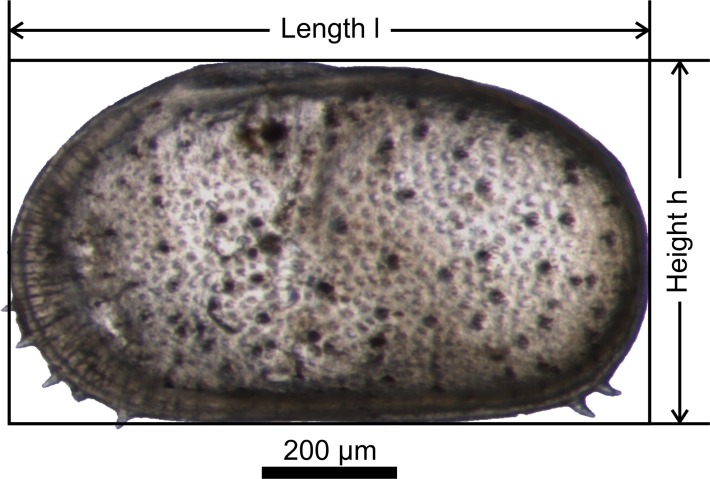
Variables measured on each valve. Left valve of a female *C*. *mataschensis* with a scheme of the variables measured on each valve.

Photographs in external view of the adult valves were taken on a Leica lightmicroscope using a Leica DFC290 camera. Maximum valve length (l) and height (h) (Fig 4) were measured using the software Leica Application Suite V3.6.0. Measurements do not include anterior and posterior spines. Analyses of the data were performed using PAST (version 3.01) [51].

**Fig 5:**
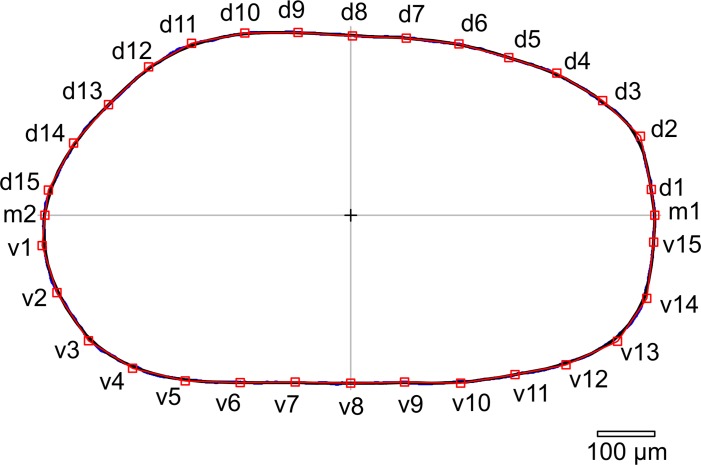
Example of the position of the control points along the outline. Digitized and reconstructed outlines of a single female left valve of *C*. *kapfensteinensis* A. m1-m2: control points on the intersection of the axis of minimum moment of inertia and the outline; d1-d15: control points along the dorsal part of the outline; v1-v15: control points along the ventral part of the outline. red: control points; blue: outline generated by tpsDig; black: outline reconstructed by MORPHOMATICA.

Outlines of 1168 *Cyprideis* valves were digitized with the TPSdig 2.14 software [52]. Geometric morphometric analyses comparing these outlines were performed with the software MORPHOMATICA 1.6 [53, 55, 54]. Outlines digitized with TPSdig are oriented in MORPHOMATICA based on their center of gravity and the main axis of inertia [53]. Outlines are then cut in half along the axis of the minimum moment of inertia avoiding random positioning of the control points [53]. MORPHOMATICA approximates the outlines using B-spline curves [46, 53]. The "control points" describing these curves can further be utilized as pseudo-landmarks [40]. We used 16 control points (i.e., 32 for the whole outline) to approximate each outline (Fig 5). The computed outlines were first compared untreated ("not normalised for area"). Later the factor size was eliminated, creating outline approximations with equal areas ("normalised for area") to evaluate differences in shape. Virtual mean outlines for each species were computed to compare general differences in size and shape of the species. Furthermore, mean outlines for each species and samples were drawn to illustrate inter- and intraspecific variances.

Differences in the total area between each outline can be translated into an Euclidean distance matrix in MORPHOMATICA [53]. Non-metric Multidimensional Scaling (nMDS) was used to visualize the disparities between the outlines [46, 53].

To maximize the variation among groups, we performed a Canonical Variate Analysis (CVA). Prior to the analysis, the coordinates of the control points were translated, rotated and scaled using the Procrustes superimposition in PAST to minimize the distance between the sets of coordinates [40].

The authors confirm that all data underlying the findings are fully available without restriction.

## Results

In total 14691 *Cyprideis* valves were picked from the fives cores with *C*. *mataschensis* being the most abundant species (7503 valves). *Cyprideis kapfensteinensis* is almost equally abundant totaling 7096 valves. Its two morphologic variations have dissimilar abundances with *C*. *kapfensteinensis* A being more abundant (6453 valves), whereas *C*. *kapfensteinensis* B is represented by 643 valves. *Cyprideis* ex gr. *pannonica* is relatively scarce with only 92 valves. All three species and both morphologic variations of *C*. *kapfensteinensis* are present with adult female and male valves as well as with up to three juvenile stages. *Cyprideis mataschensis* occurs throughout the whole high-resolution section, reaching the highest abundances (78 valves/sample at 2.34 m) slightly before the transgression of higher saline brackish waters at 2.48 m (S1 Table). The occurrence of *C*. *kapfensteinensis* A is restricted to the section between 1.76 and 3.30 m. Above 2.95 m it is only represented by a few, mostly juvenile valves. The highest abundance of *C*. *kapfensteinensis* A is recorded during the transgressive phase as well as shortly thereafter, peaking at 103 valves per sample (2.61 m). *Cyprideis kapfensteinensis* B only occurs after the transgression (above 2.48 m) and seems to be limited to salinities above 13 psu. It is missing under the meromictic conditions above 2.95 m. *Cyprideis* ex gr. *pannonica* is restricted to the part between 1.20 and 2.91 m. Its occurrence is stinted to a maximum of five valves in one sample at 2.30 m.

### Valve length

**Fig 6:**
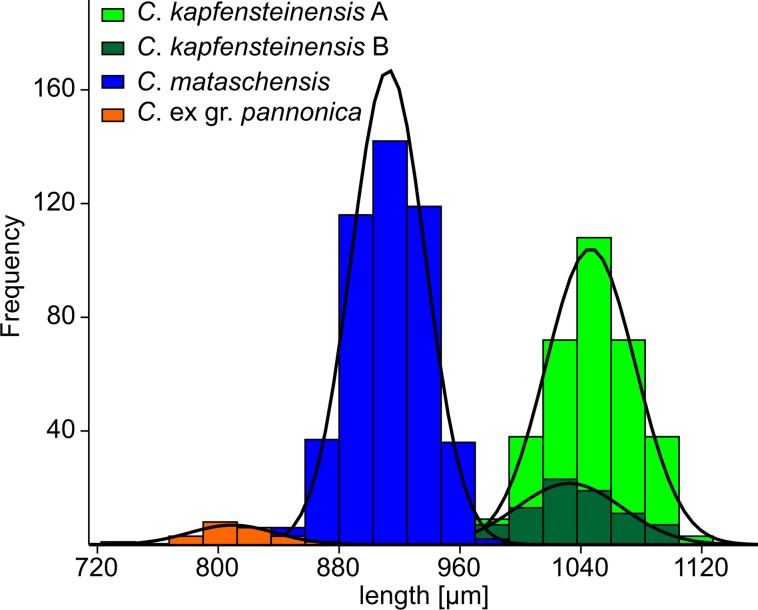
Length distributions of *Cyprideis* in Mataschen. Histograms of the length distributions of the three *Cyprideis* species and the two morphotypes of *C*. *kapfensteinensis*.

A total of 908 left and right female valves were measured for length and height (S2 Table). In each taxon, the left valves are slightly larger compared to the right valves. The 344 valves of *C*. *kapfensteinensis* A range between 965 and 1112 μm with a mean of 1046 μm (SD: 30 μm; left valves: 965–1112 μm; mean: 1059 μm; SD: 30 μm; right valves: 969–1094 μm; mean: 1034 μm; SD: 24 μm). The second morphotype *C*. *kapfensteinensis* B (84 valves) is between 952–1103 μm long, with a mean of 1032 μm (SD: 35 μm; left valves 952–1103 μm; mean: 1050 μm; SD: 34 μm; right valves: 952–1075 μm; mean: 1017 μm; SD: 28 μm). *Cyprideis mataschensis* (458 valves) has values between 842 and 972 μm with a mean length of 913 μm (SD: 25 μm; left valves: 860–972 μm; mean: 924 μm; SD: 21 μm; right valves: 842–961 μm; mean: 900 μm; SD: 22 μm). The smallest of the three species is *C*. ex gr. *pannonica* (22 valves) showing valve length between 728 and 858 μm (mean: 809 μm; SD: 29 μm). Therefore, the three species can be roughly distinguished through their individual valves length with every species showing normal probability of the measured length (Fig 6). Normal probability of the valve length is also supported through a Shapiro-Wilk test leading to high values for W of 0.9481, 0.9947, 0.9885 and 0.984 for *C*. ex gr. *pannonica*, *C*. *mataschensis*, *C*. *kapfensteinensis* A and *C*. *kapfensteinensis* B, respectively. The two morphotypes of *C*. *kapfensteinensis* are of same length.

### Outlines (not normalized)

**Fig 7:**
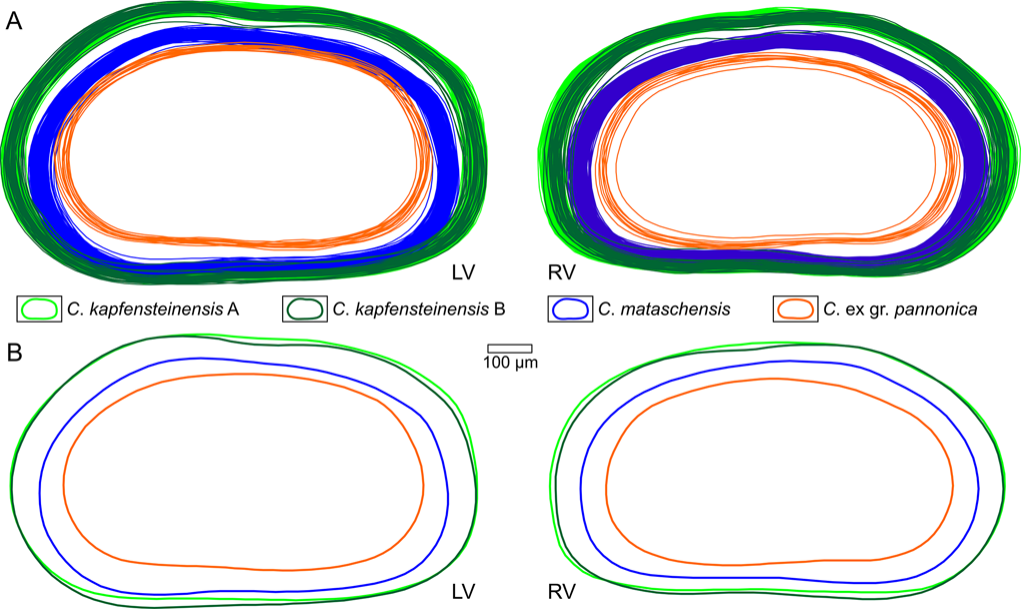
"Non-normalised" outlines of *Cyprideis* species calculated by MORPHOMATICA. a) Digitized outlines of female left and right valves. b) Mean outlines calculated for each morphotype. LV: left valves; RV: right valves.

We digitized the outlines of 444 female right valves and 349 female left valves. The 308 digitized male valves were not included in our analysis. Additionally, 67 digitized valves showed minor cracks or slight compressions and were excluded from further analysis. Outlines were computed from the lateral external view of the valves. They represent a 2D-projection of the complete outer boarder, excluding spines and denticulations. Therefore, they do not represent the shape of the contact between two valves (outer margin *sensu* [56]) as they also include the part of the "brood pouch" overlapping the contact at the posterodorsal margin. As expected by the length measurements, the outlines of the three species could be easily distinguished (Fig 7). *Cyprideis mataschensis* and *C*. *kapfensteinensis* are very similar in shape, but *C*. ex gr. *pannonica* is more elongated with the maximum height further posterior compared to the other species. Both morphotypes of *C*. *kapfensteinensis* are similar, but whereas the dorsal margin of *C*. *kapfensteinensis* A is rather straight, *C*. *kapfensteinensis* B shows a curved dorsal part, reflecting a sulcus and a more voluminous brood pouch.

**Fig 8:**
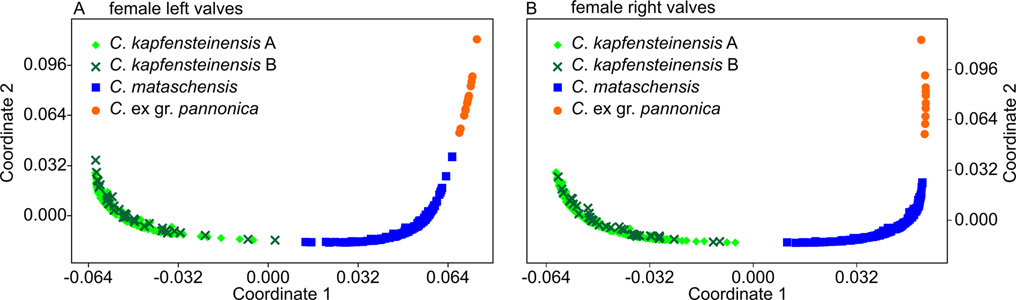
Scatter plots of the nMDS indicating the dissimilarities between the digitized outlines. a) Coordinates 1+2 for female left valves, b) Coordinates 1+2 for female right valves.

The Euclidean distance matrix obtained from MORPHOMATICA was used for nMDS. An almost perfect separation of the species is identified along the first coordinate in the left valves (Fig 8a) as well as on right valves (Fig 8b). Apart from *C*. ex gr. *pannonica*, a broad overlap along the second coordinate is observed. The two morphotypes of *C*. *kapfensteinensis* cannot be discriminated.

### Outlines normalized for area

**Fig 9:**
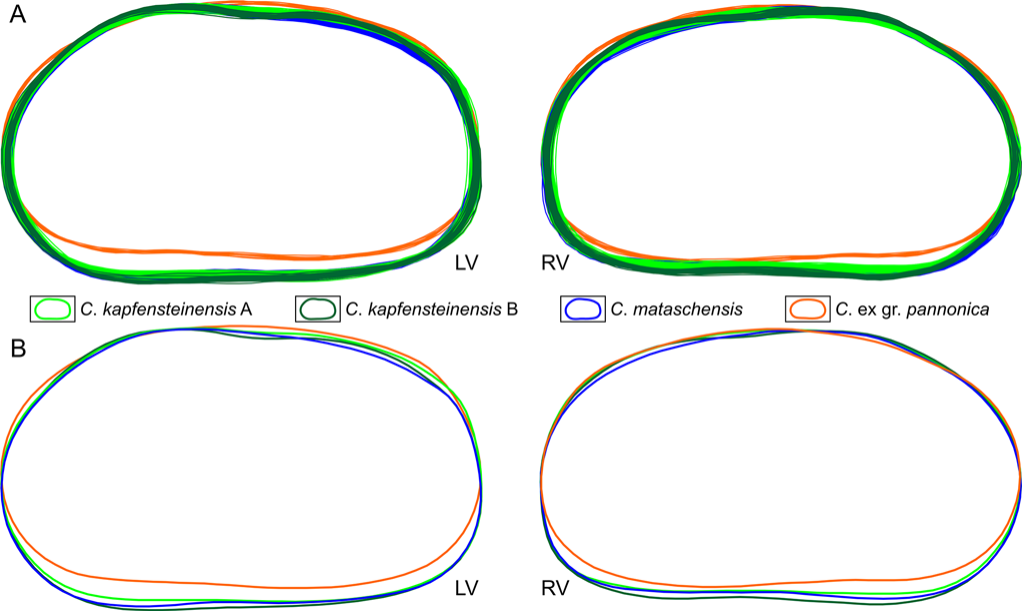
Outlines of *Cyprideis* species "normalised for area". a) Digitized outlines normalized for area. b) Calculated mean outlines of each morphotype normalized for area. LV: left valves; RV: right valves.

To test for differences in shape between the species, we normalized the outlines for their area to delete the dominating size factor (Fig 9a). Every outline is scaled to cover an area of 1 mm². From the mean outlines of each taxon a clear difference between *C*. ex gr. *pannonica* and the other two species is obvious (Fig 9b). Whereas the mean outlines indicate a small difference in the posterodorsal area between *C*. *kapfensteinensis* and *C*. *mataschensis* the intraspecific variability rejects a clear identification purely based on their outline.

**Fig 10:**
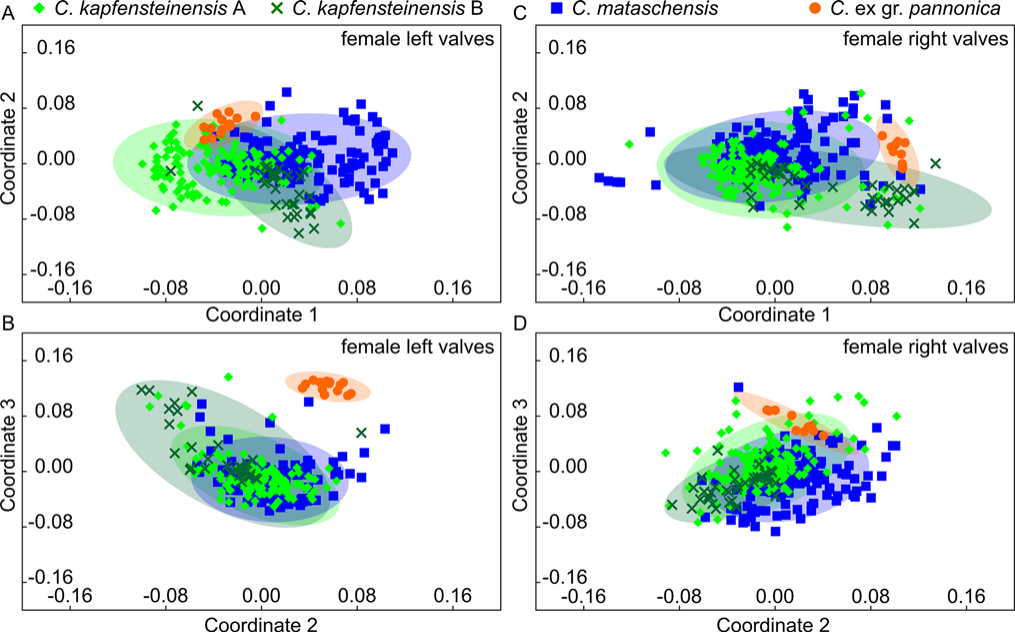
Scatter plots of the nMDS indicating the dissimilarities between the normalized outlines. a) Coordinates 1+2 for female left valves, b) Coordinates 1+3 for female left valves, c) Coordinates 1+2 for female right valves, d) Coordinates 1+3 for female right valves.

Non-metric Multidimensional Scaling supports the assessment of high intraspecific variability. In the left valves, *C*. *kapfensteinensis* A and *C*. *mataschensis* are slightly separated along the first coordinate (Fig 10a). *Cyprideis* ex gr. *pannonica* is clearly differentiated on the third coordinate from the other species (Fig 10b). The scatter plot for the right valves indicates again a broad overlap between *C*. *kapfensteinensis* A and *C*. *mataschensis*, while *C*. *kapfensteinensis* B is slightly separated along the first coordinate (Fig 10c). Along the third component a broad overlap of all species is observable (Fig 10d).

### Canonical Variate Analysis

**Fig 11:**
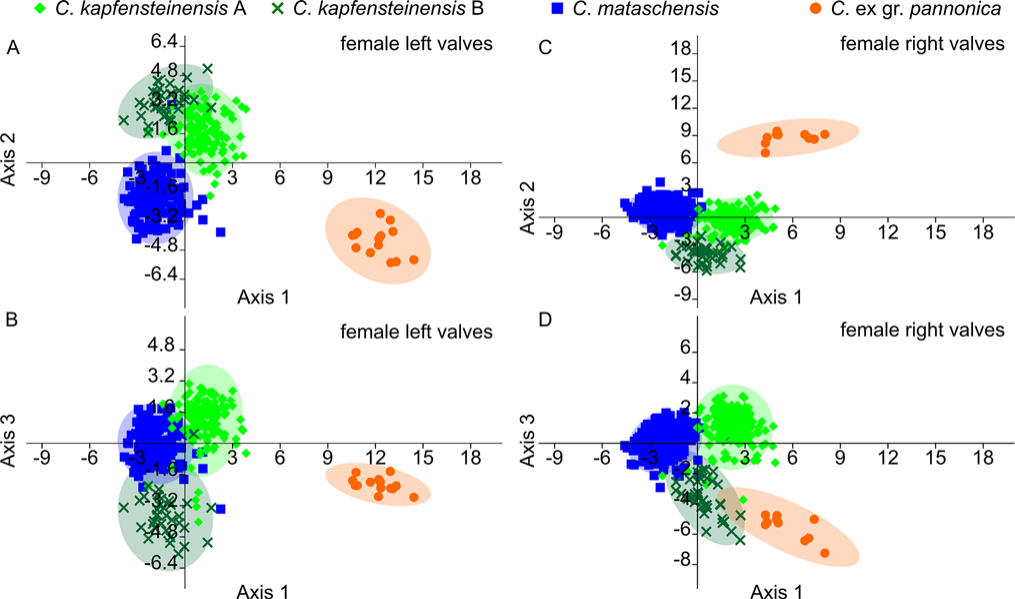
CVA plot based on pseudo-landmarks for *C*. *mataschensis*, *C*. *kapfensteinensis* A, *C*. *kapfensteinensis* B and *C*. ex gr. *pannonica*. a) Axis 1+2 for female left valves, b) Axis 1+3 for female left valves, c) Axis 1+2 for female right valves, d) Axis 1+3 for female right valves.

The coordinates of the control points for the B-spline analysis in MORPHOMATICA can be treated as pseudo-landmarks and used in a Canonical Variate Analysis to test shape differences between species and morphotypes [40]. According to the CVA, 97.71% of the left valves were correctly classified. The plot indicates a clear separation of *C*. ex gr. *pannonica* from the rest along the first axis (Fig 11a). *Cyprideis kapfensteinensis* A, *C*. *kapfensteinensis* B and *C*. *mataschensis* are slightly separated along the second and third (Fig 11b) axis. Among the right valves, 97.52% were classified correctly. Again, *C*. ex gr. *pannonica* is well differentiated (Fig 11c), while *C*. *kapfensteinens* A, *C*. *kapfensteinens* and *C*. *mataschensis* slightly overlap along all three axes (Fig 11d).

## Discussion

### Size

We measured the length of 908 valves of three *Cyprideis* species (*C*. *mataschensis*, *C*. *kapfensteinensis* and *C*. ex gr. *pannonica*) from samples of the Mataschen clay pit. These samples stem from a ~2.3 m long high-resolution interval with each 5-mm thick sample representing less than one decade [49, 57]. Ecologically, this section covers a transition from relatively shallow, slightly brackish waters of only a few meters depth to deeper and higher saline waters after a transgression. Based on dinoflagellates, salinities are estimated to be above 13 psu after the transgression [57]. Furthermore, the section comprises several intervals of depleted oxygenation and oligotrophic phases [57].

There is no overlap in size between *C*. *kapfensteinensis* and *C*. *mataschensis* within the same sample. The slight overlap we observe (Fig 6) is due to left valves of *C*. *mataschensis* being longer than right valves of *C*. *kapfensteinensis*. As in most ostracods, the left valves are generally longer than the right valves [56, 58].

Furthermore, for both species, juveniles are present at least down to A-3. The valve length between the juvenile stages of each taxon increases by a factor of 1.27–1.33 [33] and is therefore comparable to the increase of the valve length in juvenile stages of *Cyprideis torosa* [11]. Differences due to two seasonally different generations known from American *Cyprideis* [59] also seem unlikely. Kern et al [57] reconstructed a dry winter with air temperatures around 9.6–13.3°C and a wet summer with temperatures between 24.7–27.9°C for the same part of the Mataschen clay pit section. Thus, phases of increased evaporation leading to aberrant high salinities which could have had an influence on the size or shape of *Cyprideis* [18, 19] are unlikely.

**Fig 12:**
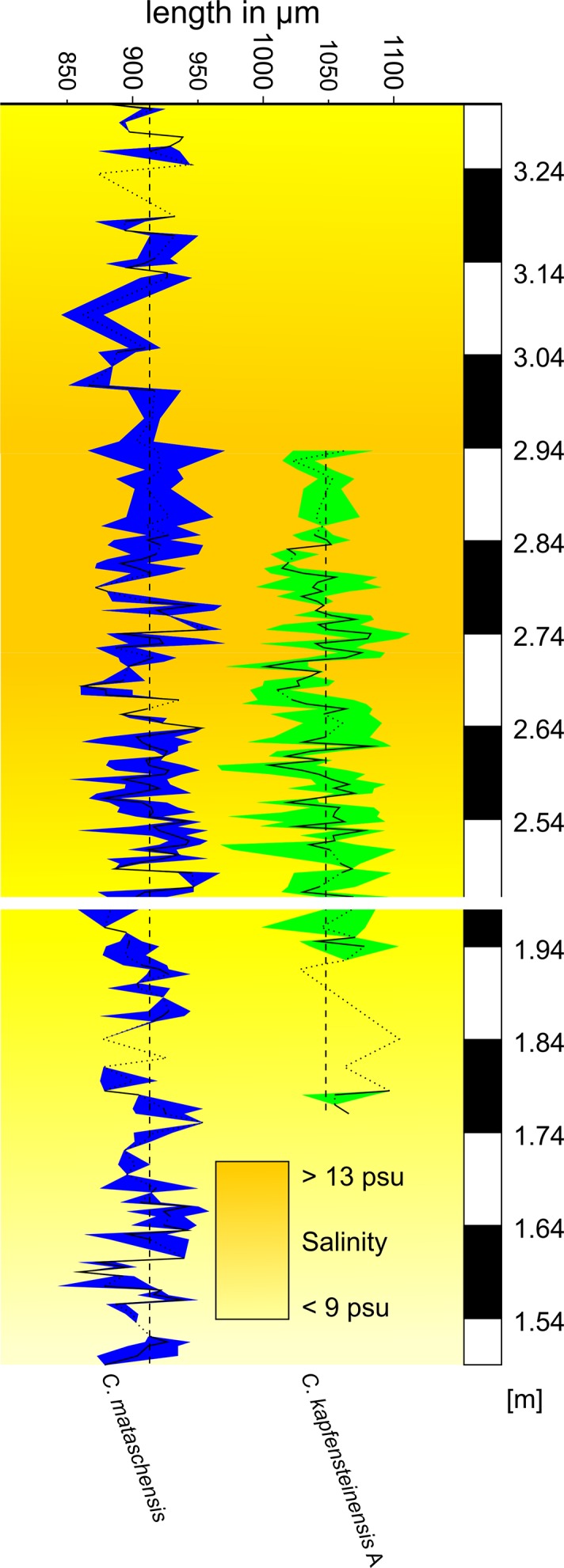
Variation in size of female *C*. *mataschensis* and *C*. *kapfensteinensis* A throughout the section. Range indicates total variation in size per sample.

Plotting length within the species along the section shows high variations within and between the samples (Fig 12). *Cyprideis mataschensis* has a maximum difference of longest *vs*. shortest valve in one sample of 104 μm (~11.4% of the mean length). The maximum difference in *C*. *kapfensteinensis* is 125 μm within one sample (11.9% of the mean length). As a general trend, the highest variability is observed in samples with high abundances. Apart from this, there is no correlation (correlation coefficient r = 0.064) between the variability of the mean length per sample of *C*. *mataschensis* and *C*. *kapfensteinensis*. Therefore, we conclude that there is either no reaction to changing environmental conditions in respect to valve length or, as there is no correlation between the variations of the two species, only one of the two species is sensitive to these changes.

Shortly after the transgression at around 2.74 m, an increase in the mean valve length of *C*. *mataschensis* corresponds to the increasing salinity above 13 psu (Fig 12). Similar mean valve lengths are recorded at the basal part of the high resolution section with low abundances and estimated salinities around 9 psu. Based on our samples, a simple linear relationship between salinity and decreasing valve length in *C*. *mataschensis* has to be rejected. Relatively short mean valve lengths for *C*. *mataschensis* are recorded between 2.64 m and 2.74 m. These samples are marked by relatively high salinities around 13 psu and relate to a recovery of the ostracod fauna after an oligotrophic phase. Another period of short mean valve length is recognized after the establishment of a meromictic lake system above 2.95 m, leading to oxygen depleted bottom waters and slightly decreasing salinities [49]. Contrary to this trend, relatively large valve sizes for *C*. *mataschensis* after a hypoxic phase at 2.75 m lead us to the conclusion that oxygen depleted conditions alone might not be related to valve length as well. We therefore infer that the length of *C*. *mataschensis* seems to be influenced not by a single, but a multitude of factors.

Likewise, *C*. *kapfensteinensis* A shows longer valve length right after their first appearance with estimated salinities around 9 psu as well as during the onset and after the transgression with estimated salinities above 13 psu. Again, no direct correlation between salinity or oxygenation and valve length can be seen. We therefore suggest that valve length of these two taxa is dependent on several factors most likely including water temperature, eutrophication, salinity, oxygenation, and most importantly genetic variability.

### Shape

Computing the mean outlines for our three species, results in a clear difference between *Cyprideis* ex gr. *pannonica*, and the two other taxa *C*. *kapfensteinensis* and *C*. *mataschensis*. *Cyprideis* ex gr. *pannonica* is more elongated with the highest point further posterior. The mean outlines of *C*. *kapfensteinensis* and *C*. *mataschensis* are very similar in shape and mainly differ in the posterior part which has a slightly steeper inclination in *C*. *mataschensis* compared to *C*. *kapfensteinensis*. Due to the high intraspecific variability, it is impossible to assign single outlines to a specific taxon. A major factor in variability is the variation in size and form of the brood pouch of the females. These variations are also known from other *Cyprideis* species [3].

The analysis of the outlines revealed differences mainly based on variations in the posterior part of the outline (Fig 9). Nevertheless, *C*. ex gr. *pannonica* is clearly separated from the other two species. Furthermore, the cluster of *C*. *kapfensteinensis* and *C*. *mataschensis* do have a different balance point and tend to scatter into slightly different directions (Fig 10). This reflects most likely the difference in the posterodorsal slope, which is more inclined in *C*. *mataschensis*.

**Fig 13:**
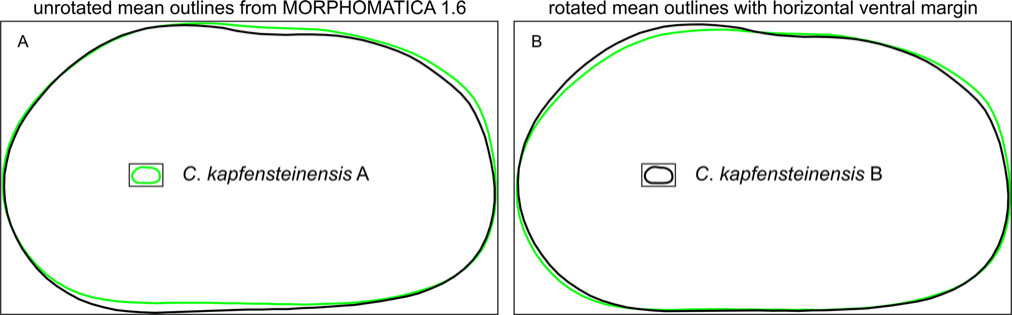
Mean outlines of *C*. *kapfensteinensis* A and B. a) Mean outlines as computed from Morphomatica 1.6, b) Mean outlines rotated to a horizontal ventral margin.

This inclination as well as the presence of a strong sulcus leads to a rotation of the outlines in MORPHOMATICA due to a shift in the centre of gravity and the axis of inertia (Fig 13a). If we rotate the outlines and align the ventral margins horizontally, we can observe that the main differences between form A and B are indeed on the dorsal margin (Fig 13b).

These differences in shape between *C*. *kapfensteinensis* A and B (sulcus) and *C*. *kapfensteinensis* A and *C*. *mataschensis* (more inclined posterodorsal slope) are supported by the Canonical Variate Analysis. While still very similar, the taxonomic classification based on different parameters (i.e., size, spines, hinge, and ornamentation) agrees well with the classification based on shape (i.e., control points as pseudo-landmarks) with more than 97% of the valves correctly assigned.

### Variations in shape related to ecology

**Fig 14:**
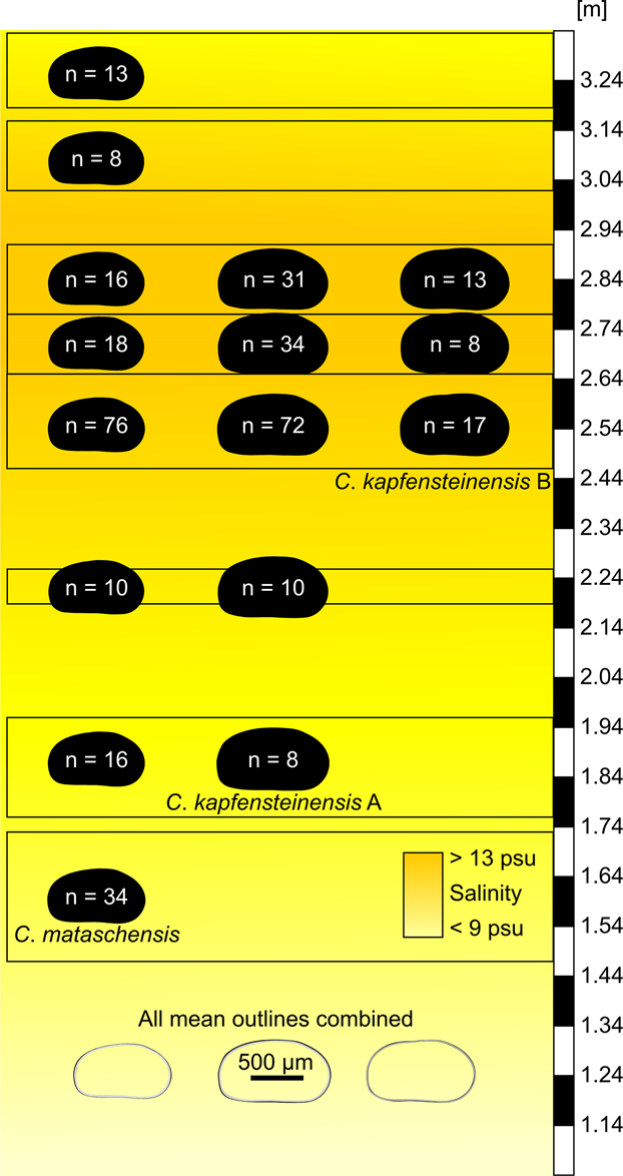
Variation in shape of female *C*. *mataschensis* and *C*. *kapfensteinensis* A and B throughout the section.

While *C*. *kapfensteinensis* is restricted to elevated salinities above approx. 9 psu and well-oxygenated bottom waters, *C*. *mataschensis* is able to cope with a wider range of salinities (<9 psu and >13 psu), comparable to recent *C*. *torosa*. To test whether *C*. *mataschensis* shows morphologic adaptations to ecologic conditions we calculated the mean outlines of female right valves from different parts of the section and compared them to the mean outline for every female right valve of the whole section (Fig 14). While the mean outline of the valves from the upper part of the section, representing a meromictic lake system with oxygen depleted bottom waters are slightly smaller, the difference is negligible compared to the overall variability.

Likewise, we compared the mean outlines from different parts of the secton of *C*. *kapfensteinensis* A to its mean outline from the whole section. As mentioned above, *C*. *kapfensteinensis* A is largest in the lowermost part of the succession, shortly after its first appearance. Nevertheless, no clear difference in shape related to diverging ecologic parameters can be found. Therefore, we conclude that the overall shape of the *C*. *mataschensis* and *C*. *kapfensteinensis* A remains stable over a wide range of ecologic parameters.

The occurrence of the second morphotype with a strong sulcus (*C*. *kapfensteinensis* B) after the transgression at salinities above 13 psu seems to be related to ecologic conditions. This adaptation to a different ecologic condition led to the development of morphologic distinct populations or even species (see below).

### Ecologic changes as trigger for speciation

**Fig 15:**
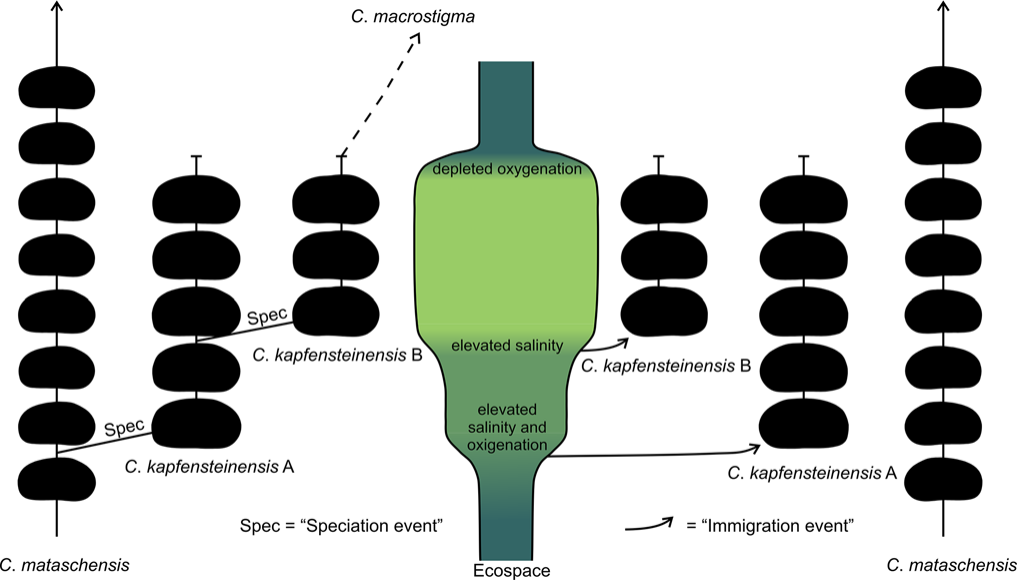
Model for the possible origins of *C*. *kapfensteinensis* A and *C*. *kapfensteinensis* B.

Currently, two possible origins of the *Cyprideis* species can be distinguished. The first occurrences of the new species/morphotypes might be related to immigration due to changing ecological conditions and/or speciation due to adaptation to a new ecological niche (Fig 15).

Within the section, several phases of changing ecologic conditions are recognized. After an initial shallow water phase (section meter 1.04–1.75 m) with lower salinity (approx. 7–10 psu) and fluctuating oxygenation, a stabilization of the environment (1.76 m) coincides with elevated salinities (approx. 9–12psu). Here, *C*. *kapfensteinensis* A has its first occurrence. Above 2.48 m, a transgression leads to salinities above 13 psu and *C*. *kapfensteinensis* B is recorded for the first time. *Cyprideis mataschensis* occurs throughout the whole section. The two first occurrences of *C*. *kapfensteinensis* A and B can be regarded either as separate immigration events of species/morphotypes, or as a morphological reaction to changing ecological conditions leading to distinct populations exploring different niches within the same ecosystem.

The first occurrence of *C*. *kapfensteinensis* A does not coincide with first occurrences of any other species (within +/- 20 cm ≈ 200 years). On the contrary, the first record of *C*. *kapfensteinensis* B is within the timeframe (+/- 4 cm ≈ 40 years) of the first occurrence of *Hemicytheria loerentheyi* (Méhes, 1908) [34] and *Callistocythere* sp. Here, a transgression of higher saline water is documented through the dinoflagellate record [57]. This emphasizes a drastic change in the environment and therefore opens the possibility to explore new ecologic niches. Nevertheless, new taxa could as well arrive with the transgression.

**Fig 16:**
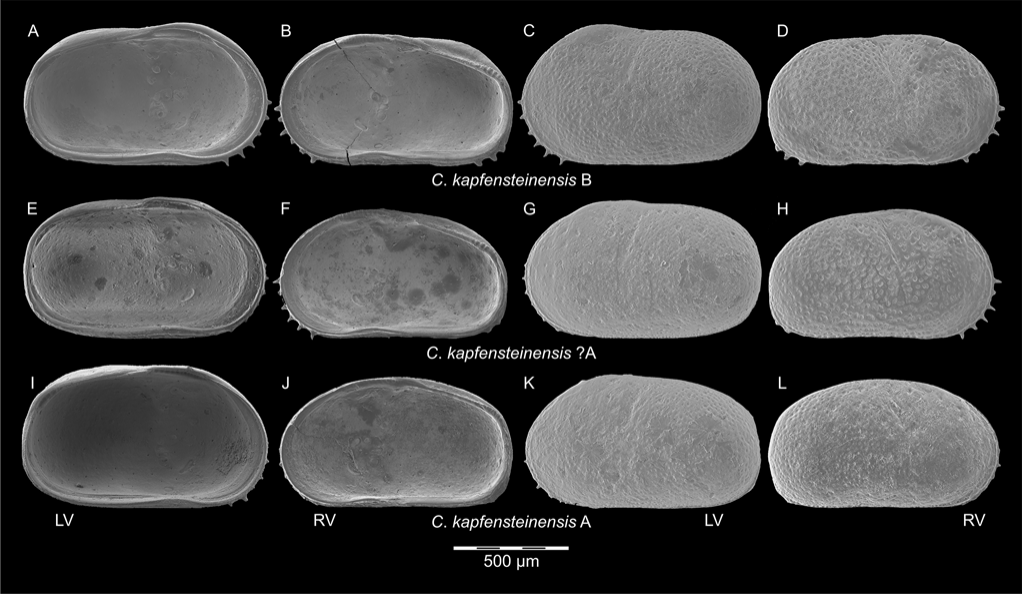
Comparison of *C*. *kapfensteinensis* A and B, and the two intermediate forms *C*. *kapfensteinensis*? A. A, C) *C*. *kapfensteinensis* B: female left valve; B, D) *C*. *kapfensteinensis* B: female right valve; E, G) *C*. *kapfensteinensis*? A: female left valve; F, H) *C*. *kapfensteinensis*? A: female right valve; I, K) *C*. *kapfensteinensis* A: female left valve; J, L) *C*. *kapfensteinensis* A: female right valve. LV: left valves; RV: right valves.

In the sample directly below the first occurrence of *C*. *kapfensteinensis* B, a single female left and a single female right valve of *C*. *kapfensteinensis* A show some kind of intermediate form between A and B. In the left valve (Fig 16g), a quite well developed sulcus hints to the general shape of form B, yet the hinge (Fig 16e) and ornamentation (Fig 16g) match form A (Fig 16i and 16k) and differs from form B (Fig 16a and 16c). The right valve (Fig 16h) has a relatively voluminous brood pouch, but its outline does not reflect the shape of form B (Fig 16d). Nevertheless, the ornamentation clearly resembles those of form B and the anterior hinge element (Fig 16f) is broader than in form A (Fig 16j), but not as broad as in form B (Fig 16b).

It is impossible to conclude whether we are observing a speciation event, or if we just see the final results of a speciation in a nearby location transported into the area via the transgression. It is certain, that *C*. *kapfensteinensis* A and B are two closely related morphotypes which might as well be seen as two distinct populations exploring different niches within the same habitat. Both populations are represented through female, male and juvenile valves (at least A-1 and A-2). Based on ornamentation, spines and outline, *C*. *kapfensteinensis* B is related to *Cyprideis macrostigma* Kollmann, 1960 [2], but there are some differences in the hinge elements. Therefore, this morphotype might be the first representative of the *C*. *macrostigma* lineage persistent in younger Lake Pannon sediments [2].

*Cyprideis torosa* is iso-osmotic at salinities >8 psu and hyper-osmotic at lower salinities. Nevertheless, this switch can already occur at 14 psu [21]. In Mataschen the first occurrence of *C*. *kapfensteinensis* A is related to salinities around 9 psu, whereas the first occurrence of *C*. *kapfensteinensis* B is related to salinities above 13 psu. It seems reasonable to speculate that physiologic adaptations to special ecologic conditions and niches might adhere to morphologic changes. Therefore, rising salinities could be a trigger for morphologic variation and as a consequence speciation in Lake Pannon *Cyprideis*.

### Speciation rates in ancient lakes

Speciation events of ostracods are well known from several long-lived lakes worldwide [22, 60–62]. Our material stems from the early phase of Lake Pannon which is known for its diverse fauna of *Cyprideis* [2, 28, 29]. At the onset of the lakes, speciation rates are expected to be much higher than during their later phases [63]. Furthermore, speciation seems to be more likely in taxa providing brood care [64]. The main difference between *C*. *kapfensteinensis* A and B is an increase of the volume of the brood pouch leading to the development of a sulcus in morphotype B. The time between first occurrence of *C*. *kapfensteinensis* A and the first occurrence of *C*. *kapfensteinensis* B in Mataschen gives us a minimum timeframe of 750 years for the speciation of *C*. *kapfensteinensis* B. This lies within the estimated speciation rates of cichlids from long-lived lakes [62].

## Conclusion

We compared the variability in size and shape of 444 female right valves and 349 female left valves of three species of *Cyprideis* from the Early Pannonian (~11.3 Ma) in the Mataschen clay pit. While two of the species, *C*. *mataschensis* and *C*. *kapfensteinensis* are very similar in shape, the third species, *C*. ex gr. *pannonica*, shows clear differences, being more elongated with the biggest height located further posterior on the dorsal margin. *Cyprideis mataschensis* and *C*. *kapfensteinensis* display a high intraspecific variability, mostly due to different developments of their brood pouch, thus making it impossible to assign outlines of a single valve to a specific taxon. Nevertheless, the mean outlines of both species are very similar, but the posterodorsal margin of *C*. *mataschensis* is slightly more inclined. The two morphotypes *C*. *kapfensteinensis* A and B can be separated based on the sulcus of *C*. *kapfensteinensis* B, leading to a "sulcate" dorsal margin compared to the rather straight dorsal margin of *C*. *kapfensteinensis* A.

Whereas *C*. *kapfensteinensis* is slightly longer during the lowest salinities, there is otherwise no clear relation between size or shape and ecologic conditions in any of the three species.

Due to their similarity in shape, but differences in ecologic preferences (generalist *vs*. specialist), we assume *C*. *mataschensis* and *C*. *kapfensteinensis* to be closely related taxa. The sub-decadal temporal resolution of the samples and the presence of intermediate forms between *C*. *kapfensteinensis* A and *C*. *kapfensteinensis* B suggest that we are in the proximity of a speciation process possibly leading to a new lineage persistent in Lake Pannon sediments.

## Supporting Information

S1 Table Total abundances of *Cyprideis* in the Mataschen clay pit. C. mat.: *C*. *mataschensis*; C. kapf.: *C*. *kapfensteinensis*; C. pan.: *C*. ex gr. *pannonica*; juv.: juvenile valves; tot.: adult + juvenile valves;? C. indet: broken valves and fragments not assigned on species level; n.s.: not sampled.
(XLSX)

S2 Table Length and height measurements on *Cyprideis* valves from the Mataschen clay pit. (XLSX)
